# Targeting Non-coding RNA for Glioblastoma Therapy: The Challenge of Overcomes the Blood-Brain Barrier

**DOI:** 10.3389/fmedt.2021.678593

**Published:** 2021-08-10

**Authors:** Rohit K. Sharma, Carlos Calderon, Pablo E. Vivas-Mejia

**Affiliations:** ^1^Comprehensive Cancer Center, University of Puerto Rico, San Juan, PR, United States; ^2^Department of Biochemistry, University of Puerto Rico, Medical Sciences Campus, San Juan, PR, United States

**Keywords:** glioblastoma, blood-brain barrier, nanoparticles, non-coding RNAs, microRNA

## Abstract

Glioblastoma (GBM) is the most malignant form of all primary brain tumors, and it is responsible for around 200,000 deaths each year worldwide. The standard therapy for GBM treatment includes surgical resection followed by temozolomide-based chemotherapy and/or radiotherapy. With this treatment, the median survival rate of GBM patients is only 15 months after its initial diagnosis. Therefore, novel and better treatment modalities for GBM treatment are urgently needed. Mounting evidence indicates that non-coding RNAs (ncRNAs) have critical roles as regulators of gene expression. Long non-coding RNAs (lncRNAs) and microRNAs (miRNAs) are among the most studied ncRNAs in health and disease. Dysregulation of ncRNAs is observed in virtually all tumor types, including GBMs. Several dysregulated miRNAs and lncRNAs have been identified in GBM cell lines and GBM tumor samples. Some of them have been proposed as diagnostic and prognostic markers, and as targets for GBM treatment. Most ncRNA-based therapies use oligonucleotide RNA molecules which are normally of short life in circulation. Nanoparticles (NPs) have been designed to increase the half-life of oligonucleotide RNAs. An additional challenge faced not only by RNA oligonucleotides but for therapies designed for brain-related conditions, is the presence of the blood-brain barrier (BBB). The BBB is the anatomical barrier that protects the brain from undesirable agents. Although some NPs have been derivatized at their surface to cross the BBB, optimal NPs to deliver oligonucleotide RNA into GBM cells in the brain are currently unavailable. In this review, we describe first the current treatments for GBM therapy. Next, we discuss the most relevant miRNAs and lncRNAs suggested as targets for GBM therapy. Then, we compare the current drug delivery systems (nanocarriers/NPs) for RNA oligonucleotide delivery, the challenges faced to send drugs through the BBB, and the strategies to overcome this barrier. Finally, we categorize the critical points where research should be the focus in order to design optimal NPs for drug delivery into the brain; and thus move the Oligonucleotide RNA-based therapies from the bench to the clinical setting.

## Introduction

GBM is the most common and aggressive malignant brain tumor in adults accounting for around 48% of all primary malignant brain tumors. The majority (90%) of GBMs develop *de novo* (primary glioblastoma) and 10% progress from lower astrocytomas. Most GBM occurs spontaneously, although familial gliomas have also been noted (1). The lethality of GBMs is mostly due to the ability of cancerous cells to spread over the brain parenchyma and its high level of vascularization (2). Evidence has shown that the mean survival rate for diagnosed GBM is 8–15 months and for recurring GBM the mean survival rate is 3–9 months. The 5-year survival rate is only 5% (3). The standard treatment for GBM patients includes surgery (to remove tumors, when possible) combined with radiotherapy (RT) and/or temozolomide (TMZ)-based chemotherapy (4). TMZ is a prodrug, which converts into its active metabolite via a non-enzymatic pathway (5). The active metabolite of TMZ targets guanine at the position of O6 and N7, preventing DNA replication and inducing apoptosis (6). Data from the European and Canadian trial (EORTC 26981/22981-NCIC) demonstrated that RT, followed by 6 monthly cycles of TMZ provides significant survival benefits with minimal additional toxicity in patients with GBM. They reported a median survival of 14.6 months with RT plus TMZ *vs*. 12.1 months with RT alone (6–8).

Other FDA approved treatment modalities for GBM treatment include bevacizumab (Avastin) an antibody against the Vascular Endothelial Growth Factor Receptor (VEGFR), carmustine (BCNU) also known as gliadel wafers (4, 8). The latter are composed of nitrosoureas which alkylate DNA by forming inter-strand crosslinks to prevent DNA from replication or transcription when implanted in the cavity of the resected tumor (9), lomustine, another alkylating agent of the nitrosourea type is highly lipid-soluble and able to crosses the BBB (10). Several therapeutic modalities for GBM in clinical trials include monoclonal antibodies targeting Epidermal Growth Factor Receptor (EGFR) (alone or conjugated with other drugs), chemotherapeutic agents (procarbazines, hydroxyureas, irinotecan, erlotinib, cyclophosphamide, and mTOR inhibitor), tumor treating fields (TTF) therapy, immunotherapies (vaccination, adoptive cell therapy, and immunostimulants), epigenetic therapy, oncolytic virus therapy, and gene therapy (11, 12). Despite all available diagnostic, prognostic, and therapeutic modalities, the GBM prognosis remains poor. It has been speculated that tumor heterogeneity and the presence of cancer stem cells in the tumors are major reasons for therapy resistance of GBM cells (13). Therefore, novel therapeutic approaches are needed to improve the survival and quality of life for GBM patients.

In the last two decades, ncRNAs have gained significance as potential targets against many cancer types, including GBMs (14). NcRNAs represent more than 60% of the human genome (15), and based on their length they are usually divided into two major types, small non-coding RNAs (sncRNAs), and long non-coding RNAs (lncRNAs). ncRNAs can also be divided into structural and regulatory functions. Structural ncRNA includes t-RNA, r-RNA, snRNAs, and snoRNAs, among others (16). Regulatory ncRNA includes mainly, the Xist (the ncRNA responsible for female X chromosome inactivation early during embryogenesis), the microRNAs (miRNAs), and lncRNAs. Although the role of miRNAs as posttranscriptional regulators of gene expression has been well-documented, the multiple potential roles of lncRNAs are presently under extensive investigation. In any case, dysregulation of miRNAs and ncRNAs in most cancer types including GBMs and therefore, these molecules have been considered as diagnostic, prognostic and therapeutic tools. MiRNA-based therapies are designed to target upregulated miRNAs with oligonucleotide miRNA inhibitors (OMIs) or against downregulated miRNAs with oligonucleotide miRNA mimics (OMMs) (17). LncRNAs and mRNA-coding genes are targeted with double-stranded RNAs (small interference RNA, siRNA). OMIs, OMMs and siRNA-based molecules are all referred to as interference RNA (RNAi) (17).

In the next sections, we discuss the current status of the RNAi-based therapies under investigation for GBM treatment, the proposed delivery methods for drug delivery into the brain, and finally, we address the research status of the strategies to overcomes the BBB for GBM treatment.

## Deregulation of Non-Coding RNAS in GBM

After their discovery in *Caenorhabditis elegans* by Lee et al. (18) miRNAs were soon associated with the regulation of gene expression at the posttranscriptional (mRNA) level. Early expression profile studies showed deregulation of several miRNAs in many diseases including cancer (19, 20). Similarly, Brannan et al. in 1990 reported for the first time H19 as a lncRNA (21), and in 1991 Brown et al. reported the lncRNA Xist as responsible for the X-chromosome inactivation (22). All these efforts along with the sequencing of the human genome in 2001 made clear that close to 80%) of the DNA encode ncRNAs. Further evidence confirmed that ncRNAs play a central role in the regulation of gene expression (23, 24). Remarkably, for Xist and other lncRNAs, such as in Air, only small segments in their sequences are highly conserved (25, 26). For instance, nearly 5% of mammalian lincRNAs (long intergenic non-coding RNAs that constitute more than half of lncRNAs transcripts in humans), which are typically restricted to short polynucleotide stretches, are conserved in zebrafish (27). Some mouse and human lncRNAs orthologs were able to phenotypically rescue zebrafish lincRNA loss of function indicating that at least some lincRNAs are functionally conserved across species (26).

Unlike lncRNAs, miRNAs are well-conserved across a diverse range of species (15). It is thought that due to the short size of the miRNA molecules, any change in their sequences will result in major effects on their functions (25). McCreight et al. analyzed the miRNA sequences of 13 primate species (Apes, Old World monkeys, New World monkeys, and Strepsirrhines) and observed that not only the seed region and the mature miRNA but also the pre-miRNA hairpin sequences are highly conserved across primates (28).

Several studies have confirmed the relationship between ncRNAs expression and human diseases including cancer (29–31); neurological and neurodegenerative diseases such as Alzheimer's, Schizophrenia, Autism spectrum disorder, Parkinson's, Angelman syndrome, Huntington's disease among others (30). Deregulation of ncRNAs has also been associated with diseases related to endocrinology, reproduction, metabolism, immunology, neurobiology, muscle biology, and cancer (32).

### MicroRNAs

MiRNAs are endogenous sncRNAs of around 22–24 nucleotides long which regulate gene expression at the posttranscriptional level by binding mainly to the 3′ untranslated (3' UTR) regions of mRNAs (33). The genesis of miRNAs has been extensively described in the literature (34). Several expression profile studies using cell lines and tumor samples have identified many dysregulated miRNAs in GBM cell lines and GBM tumor samples compared with normal control samples (35, 36). Deregulation of those miRNAs could play important role in all steps of GBM initiation, progression, and tumor maintenance (37). Some of these miRNAs and their downstream molecular have been proposed as targets for GBM therapy (36, 38).

MiR-21, one of the first discovered miRNAs was shown to exhibit oncogenic properties (oncomiR) (39). MiR-21 regulates several tumor suppressor genes including PTEN, TIMP3, PDCD4, among others (39). Corsten et al. reported that suppression of miR-21 with locked nucleic acid (LNA)-antimiR-21 oligonucleotides increased caspase activity and decreased cell viability of human GBM cells (A172 and U-87 MG). LNA against miR-21 also had beneficial effects on intracranially implanted GBM mouse models (40). Inhibition of miR-21 expression in T98G human GBM cells with catalytic nucleic acids (hammerhead ribozymes and DNAzymes) increased PTEN expression and decreased cell proliferation and invasion (41). Inhibition of miR-21 also increased apoptosis and sensitized chemo- or radiotherapy-resistant cells to other treatments (41). Piwecka et al. performed miRNA expression studies using miRNA microarrays, deep sequencing, and meta-analysis in GBM and peritumoral brain tissues obtained from the patients during surgery and compared their findings with normal brain tissues (42). Their findings identified 35 miRNAs which were either upregulated or downregulated in GBM *vs*. control samples. They proposed 30 of these miRNAs as novel biomarkers for GBM (42).

By using COX regression analysis, Srinivasan et al. identified 10 significant dysregulated miRNAs in GBM patients compared with normal brain samples (43). These miRNA signatures were able to discriminate between patients with high vs. low survival rates (43). Similarly, Jin and workers proposed a novel method for the prioritization of candidate cancer-related miRNAs which alter the expression of other miRNAs and coding genes across an entire biological network (44). To do this, they selected three important features: the average expression of a miRNA in multiple cancer samples, the average of the absolute correlation values between the expression of a miRNA and expression of coding genes, and the number of predicted miRNA target genes. The clinical relevance of the top 20 miRNAs of this study was interrogated using microarray and/or RNA-Seq datasets available in “The Cancer Genome Atlas” (TCGA) data portal (http://cancergenome.nih.gov) (44). MiR-22 emerged as the top relevant miRNA in GBM (44). An additional study reported that miR-22 was downregulated in GBM tissue samples and GBM cell lines as compared with non-tumor tissues and normal human astrocytes, respectively (45). MiR-22 downregulation correlates with the upregulation of SIRT1 (NAD-dependent deacetylase sirtuin-1), a gene associated with the repression of p53-mediated apoptosis (46). Therefore, therapies increasing the miR-22 levels could have beneficial effects in GBM patients (45, 46).

In the most recent work, Boissinot et al. performed a high-throughput screen study in adult (U251) and pediatric GBM cells (KNS42) using a synthetic oligonucleotide library that mimics the annotated mature miRNAs (miRBase v16.0) and measured the reduction of cell proliferation in the cell lines following transfection of the mimics. This screening identified ~100 significantly cytotoxic miRNAs. Mir-1300 was shortlisted as the most potent and robust candidate miRNA. Further experiments revealed that ectopic expression of miR-1300 decreased tumor growth in an orthotopic U-87 MG mouse model, indicating that miR-1300 is a potential candidate for therapeutic applications (47). Recently, miR-29a, a miRNA with tumor suppressor capabilities, was reported downregulated in GBM cells (U-251 MG, U-87 MG, U-373 MG, U-1242 MG, and T98G), glioma stem cells (GSCs) (GSC-11, GSC-20, GSC-28, GSC-267, GSC-295, and GSC-627), and human GBM tumors samples as compared with normal human astrocytes (NHAs) and normal brain tissues, respectively (48). When miR-29a was exogenously expressed in GBM (U-87 MG) and GSCs (GSC267) cells, it induced apoptosis and inhibited cell growth, migration and invasion (48). In addition to miR-29a, miR-370 was found to be downregulated in human glioma tissue samples, U-87 MG and U-251 MG cells as compared with control samples. Decreased levels of miR-370 have been associated with the malignant transformation of astrocytes into glioblastomas and astrocytomas (49). Transfection of OMIs or OMMs in U-87 MG and U-251 MG cells showed that downregulation of miR-370 is negatively associated with β-catenin (upregulation) and positively linked with nuclear FOXO3a (forkhead box protein) (49). β-catenin is a downstream molecule of the Wnt and Hedgehog signaling, the main pathway of GSCs renewal (50). Additionally, miR-320 was decreased while forkhead box protein M1 (FoxM1) was increased in radioresistant glioma tissues obtained from GBM patients (51). The direct binding of miR-320 to FoxM1 was confirmed by bioinformatics and luciferase reporter assays (51). MiR-320 overexpression in U 251 MG and U-87 MG cells followed by infrared exposure, reduced cell survival, and increased apoptosis compared with controls (51). These findings also showed that miR-320 improves the radiosensitivity of glioma cells via down-regulation of Sirt1.

Collectively, numerous studies have demonstrated that several miRNAs are deregulated in GBM cell lines and GBM tumor samples. The selection of appropriate miRNAs as a target for GBM therapy (or any other disease) should identify the precise miRNA target genes as a single miRNA can regulate tumor suppressors and/or oncogenes at the same time (52). Similarly, a gene can be regulated by more than one miRNA (53). During the course of the disease, the expression of a particular miRNA may change, especially, in cancer stem cell populations, drug-resistant cell populations, or in response to therapy (radiotherapy and/or chemotherapy). Also, the expression of a miRNA may be different (even opposite) in GBM cancer cells *vs*. the tumor microenvironment. Table 1 summarizes some relevant miRNAs that have been targeted with OMIs or OMMs in GBM mouse models.

**Table 1:** List of most relevant MiRNAs as potential targets against GBM.

**MicroRNA**	**Up-or-down regulated**	**Biological role**	**GBM model and therapy delivery method**	**References**
miR-10b	Upregulated	OncomiR, promotes proliferation of GBM and GSCs	Human GSC (GBM8) and mouse GL261 cells implanted InCr. Therapy: anti-miR IT, IV, and with osmotic pumps.	(54)
miR-486-5p	Upregulated	OncomiR, enhances the survival of GBM stem cells	Neurosphere-derived xenografts (GBM1A and Mayo39 cells) implanted InCr. Therapy: nano formulation of anti-miRs, InCr	(55)
miR-21-5p	Upregulated	OncomiR, promotes tumor cell survival and invasiveness, involved in TMZ resistance	U-251 MG cells implanted SC Therapy: antisense miR- 21/oligofectamine, IT	(56)
miR-34a	Downregulated	Tumor Suppressor, modulates EGFR	U-87 MG cells implanted SC and InCr. Therapy: miR-34 as polyglycerol-based polyplex formulation, IT and IV	(57)
miR-128-3p	Downregulated	Tumor suppressor, inhibits metastasis and epithelial-mesenchymal transition	U-251GM cells implanted SC Therapy: LV-miR +/- TMZ: IP	(58)
miR-143-3p	Upregulated	OncomiR, increase cell proliferation	U-87 GM cells implanted SC. Therapy: liposomal anti-miR, IP	(59)
miR-148a/miR-296-5p	Controversial	OncomiR or Tumor suppressor	Human GBM derived neurospheres (GBM1A) implanted InCr. Therapy: PBAE nano-miRs mimics NPs, InCr	(55)

*InCr, intracranial; IT, intratumoral; IP, intraperitoneal; IV, intravenously; NPs, nanoparticles*.

### Long Non-coding RNAs

LncRNAs are RNAs longer than 200 nucleotides that are not translated into proteins (32). Based on their genomic localization, orientation, and processing lncRNAs are divided into at least five categories: intergenic, intronic, bidirectional, and antisense lncRNAs (60). Classification of lncRNAs according to their function, is possible (61, 62). A study by Ma et al. revealed that the human genome expresses at least 270,044 lncRNAs (63). LncRNAs play important roles in the regulation of various biological processes in the nucleus, cytoplasm, and even in the extracellular space by mechanisms that include lncRNA-protein, lncRNA-RNA, lncRNA-miRNA, and lncRNA-DNA interactions (64). Moreover, evidence indicates that lncRNAs play a central role in other molecular events including regulation of mRNA stability, RNA splicing, chromatin structure, miRNA mediated gene regulation, protein, and enzyme function, and as extracellular signaling molecules (65). Similar to miRNAs, deregulation of lncRNAs includes, duplications/translocations/mutations in DNA sequences coding the lncRNAs, alteration in signaling molecules and transcription factors responsible for the lncRNAs regulation, modifications in the RNA recognition sites, and alterations in the levels of molecules interacting with a particular lncRNAs (66). However, the entire biological roles and molecular mechanisms of lncRNAs are still under investigation.

Deregulation of lncRNAs has been associated with all steps of carcinogenesis in most cancer types, including GBM (67). For example, the lncRNA LINC00152 is upregulated in GBM patients and was correlated with poor prognosis (68, 69). SiRNA-mediated LINC00152 silencing suppressed tumor growth and invasion in both *in vitro* and in an intracranial GBM (U87-MG cells) mouse model (68). Further experiments showed that LINC00152 regulates a miR-612/AKT2/NF-κB pathway to promote proneural–mesenchymal transition (PMT) (68). A recent study using available clinical and molecular GBM patient information and bioinformatics tools identified 10 lncRNAs that can be used as an independent prognostic factor for high-grade gliomas (70). Li et al. reported that an extremely poor overall survival of GBM patients was linked to the upregulation of the linc00645 lncRNA (71). ShRNA-mediated knockdown of linc00645 in U-251 MG and T98G GBM cells inhibited colony formation, invasion, and migration while the self-renewal ability (neurosphere formation) of these cells was reduced (71). *In vivo* studies where Linc00645 was knocked-out in U-251 MG-Luc cells and intracranially injected nude mice reduced the tumor growth compared with a lncRNA control (71). Further molecular signaling pathway analysis showed that linc00645 targets the miR-205-3/Zinc Finger E-box-Binding Homeobox 1 (ZEB1) pathway (71). In other studies, Ren et al. demonstrated that siRNA knock-down of the lncRNA SNHG7 (small nucleolar RNA host gene 7, a lncRNA upregulated in several cancers, including GBM tissues and cell lines) in A172 and U-87 MG cells reduced cell proliferation, migration and invasion, and activated apoptosis. SiRNA-mediated SNHG7 knockdown also reduced tumor growth and metastasis in GBM xenograft (*s.c*.) mouse model (72). Ren et al. proposed that SNHG7 directly inhibits miR-5095 and activates the Wnt/βcatenin signaling pathway. Additionally, inhibition of miR-5095 in A172 and U-87 MG cells increased the expression of CTNNB1, the gene encoding β-catenin (72). More recently, Chen et al. reported that SNHG7 acts as a miRNA sponge by reducing miRNA-449b-5p levels, increasing MYCN (a miRNA-449b-5p target gene), and promoting GBM progression (73). In other studies, Han et al. reported that the Wnt/β-catenin signaling pathway in GBM is inhibited by the lncRNA MIR22HG through the loss of miR-22-3p and miR-22-5p (74). Furthermore, MIR22HG was highly abundant in GBM patient samples and its expression correlated with poor prognosis. This research team also identified SFRP2 and PCDH15 as direct targets of miR-22-3p and miR-22-5p in GBM cells. Additionally, they designed a small inhibitor (AC1L6JTK) inhibited tumor growth in *s.c*. GBM mouse model (74).

Another lncRNA that is increased in GBM tumor samples compared to normal brain tissues is the NF-kappa B interacting lncRNA (NKILA). High levels of NKILA correlated with reduced GBM patient survival (75). An shRNA containing lentiviral vector against NKILA showed that this lncRNA stimulates the activity of the hypoxia signaling pathway, the Warburg effect, and angiogenesis in GBM cells (75). These effects were reversed when 20(S)-Rg3 monomers (an anti-cancer agent and apoptotic inducer that interfere with multiple signaling pathways) (76) were injected *s.c*. in LN229 or U-87 MG tumor-bearing mice. Furthermore, 20 (S)-Rg3 inhibited the expression of NKILA and reversed the stimulation of the Warburg effect and angiogenesis in LN229 and T98G glioma cells (75).

Many reports have shown a direct correlation between lncRNA expression levels and TMZ resistance (77–80). For instance, Cai et al. showed that the lncRNA MALAT1 was significantly upregulated in TMZ-resistant U251 GBM cells. Lentiviral-based knockdown of MALAT1 decreased TMZ resistance in GBM cells as confirmed by the reduction of cell growth and increased apoptotic rates (77). A significant reduction in tumor volume and tumor weight was observed when U251/TMZ cells were injected *s.c*. in nude mice. Further experiments indicated MALAT1 suppressed the expression of miR-101, suggesting a potential mechanism for MALATI in TMZ resistance (77). In separate experiments, Wu et al. performed a lncRNA microarray of RNA extracted from patient-derived LN229 cells and its TMZ resistant counterpart 229R GBM cells. The microarray identified lnc-TALC (temozolomide-associated lncRNA in glioblastoma recurrence) as one of the most abundant LncRNAs that was expressed differently between the two cell types. CRISPR-Cas9-mediated knockdown of lnc-TALC significantly decreased cell viability, promoted cell apoptosis, and inhibited colony formation and proliferation following TMZ treatment of TMZ- resistant GBM cells. By contrast, overexpression of lnc-TALC in LN229 and HG7 GBM cells reduced apoptosis and increased cell proliferation and colony formation after TMZ treatment. Tomography studies in mice implanted intracranially with LN229 and 229R cells revealed that TMZ resistant tumors did not respond to TMZ treatment while knockdown of lnc-TALC restored the sensitivity to the drug (78). Further evidence showed that lnc-TALC binds to miR-20b-3p with the concomitant expression of c-Met (78).

Similarly, it has been found that levels of the lncRNA SOX2OT are higher in TMZ resistant cells and recurrent GBM patient samples compared with normal human astrocytes (NHA). Knockdown of SOX2OT suppressed cell proliferation, promoted apoptosis, and enhanced TMZ sensitivity (79). Further studies showed that TMZ resistance was achieved by the interaction of SOX2OT with ALKBH5, a mammalian dioxygenase that oxidatively demethylates m (6) A in the mRNA (79). Recent studies confirmed that knockout of ALKBH5 enhanced the efficiency of immunotherapy and prolonged survival of mouse pre-implanted with B16 mouse melanoma or CT26 colorectal carcinoma cells (81). Mazor et al., using mathematical models to analyze multiple molecular pathways at the same time (82), RNA sequencing, and gene expression profiling datasets identified TP73-AS1 lncRNA as overexpressed in primary GBM samples and GSCs compared with normal brain tissues. To clarify the role of this lncRNA, the authors of this study performed a viral infection of a CRISPR interference (CRISPRi) targeting TP73-AS1. Results indicated that TP73-AS1 enhances TMZ resistance by promoting the expression of ALDH1A1 (aldehyde dehydrogenase 1 family member A1) (82). ALDH1A1 is a well-known marker of cancer stem cells and a drug resistance promoter in cancer cells (82).

Together, several dysregulated lncRNAs have been identified in GBM cell lines and GBM tumor samples, including TMZ resistant cells. More studies are needed to clarify the role of lncRNA in GBM initiation, progression, and drug resistance. Additional, therapeutic experiments using orthotopic and patient-derived GBM cells are required to confirm the beneficial effects of targeting specific lncRNAs in GBM tumors. Table 2 summarizes lncRNA that have been targeted with siRNAs or small molecule inhibitors in GBM mouse models.

**Table 2:** List of relevant deregulated lncRNAs proposed as therapeutic targets against GBM (GBM mouse models used in the study).

**LncRNA**	**Up-or-down regulated**	**Biological role**	**Mouse model used for therapy**	**References**
SNHG7	Upregulated	Inhibition of miR-5095 and activation of Wnt/β-catenin signaling pathway.	xenograft experiments in nude mice	(72)
MALT1	Upregulated	EGFR-induced NF-κB activation	U-87 MG cells implanted InCr Therapy: MI-2 small molecule inhibitor, IP	(83)
lncRNA- TALC	Upregulated in TMZ resistant cells	Promotes O6-methylguanine- DNA methyltransferase expression, TMZ resistance	GBM cells (LN229/229R and 229R Scra/229R KD_lnc) implanted InCr. Therapy: TMZ, IP	(78)
SOX2OT	Upregulated in TMZ resistant cells	IncreasesSOX2 expression and activate the Wnt5a/β-catenin signaling pathway, TMZ resistance	U87TR-sh-NC and U87TR-sh-SOX2OT cells implanted SC. Therapy: TMZ, IP	(79)
MIR22HG	Upregulated	Inducer of the Wnt/β-catenin signaling pathway	U-87MG cells implanted SC. Therapy: AC1L6JTK small-molecule inhibitor, IP	(74)

*InCr, intracranial; IT, intratumoral; IP, intraperitoneal; IV, intravenously; NPs, nanoparticles*.

## Delivery Strategies for RNAi-Based Therapies Against GBM

Several factors limit the use of RNAi-based therapy for cancer treatment. RNAi-based molecules are rapidly degraded in the circulation and cleared by the renal system. They activate immune responses, and due to their negative charge, they are not able to cross the cell membrane (84). Part of these obstacles has been solved using NPs which extend the half-life of RNAi molecules in the circulation. However, once inside cells, NPs-RNAi are entrapped in the endolysosomal pathway and degraded (84).

Many NPs have been proposed for the systemic delivery of RNAi-based molecules, including liposomes, polymeric NPs, micelles, dendrimers, artificial DNA nanostructures, silica NPs, nanotubes, metal NPs (mostly, Fe-, Au, and Se-based), and quantum dots, among many others (85–90). Although all of these NPs have been proposed as drug carriers for cancer treatment, few have been tested in relevant animal models of cancer. NPs are modified with ligands to increase their stability in serum (i.e., using polyethylene glycols, PEG) and/or to improve the endosomal escape (i.e., with polyethylenimine, PEI) (passive targeting). Depending on the construction material, NPs are derivatized with peptides, antibodies, carbohydrates, or other cell or tissue-specific markers (active targeting) (84, 91, 92). Important parameters to take into account with NPs for cancer treatment are their size, charge, and biodegradability (in particular, metal NPs accumulate in the organs and are therefore toxic) (90, 93–97). It is speculated that 50–200 nm in diameter are an ideal NP size for effective retention on the tumor tissues, and 20–50 nm for drug delivery through the BBB (90, 98) (see section Viral-Based Delivery for the discussion of RNAi delivery through the BBB). In terms of the charge, positively charged NPs enter easily inside cells and therefore are more toxic compared with negatively charged or neutral NPs (97, 99). However, positively charged NPs are more rapidly phagocytized by the mononuclear phagocyte system which negatively impacts their accumulation in the tumors (100). Also„ the NP surface is cover with different proteins which could change their size and their surface characteristics as they travel in the circulation and organs (101). These modifications (protein corona) are not well-investigated.

PEI-coated Fe_3_O_4_ NPs with siRNA were used for the silencing of the repressor element 1-silencing transcription factor (REST) gene in U-87 MG and U-251 GBM cells. The inhibition of REST significantly decreased cell viability and migration in these cells (90). Qui et al. effectively silenced Bcl-2 and VEGF in U-87 MG cells when siRNAs were delivered as a complex with β-cyclodextrin (β-CD)-modified dendrimer-entrapped gold nanoparticles (Au DENPs) (102). More recently, Li et al. proposed a composite NP of cationic liposomes loaded with YAP (yes-associated protein 1)-targeting siRNA and doxorubicin and coated on the exterior with gold nanorods. These NPs silenced YAP in GBM cells and reduced tumor growth when systemically injected in orthotopic GBM mouse models (103). Currently, most of these metal NPs as well as liposomes, polymeric NPs, and other composites are used in combination (i.e., gold NPs and liposomes) and/or modified with ligands to improve their stability and specificity (see below).

### Liposomes and Polymeric NPs

Liposomes are artificial phospholipid vesicles that form a “core-shell” structure and can be readily loaded with therapeutic agents (104). Lipids commonly used for liposome preparation are biocompatible, biodegradable, and of low toxicity. Liposomes increase the distribution of delivered agents, reduce toxicity, and extend half-life. Liposomes can be customized based on the feasibility of the target cell or tissue by modifying their surfaces with a variety of functional moieties (17, 105, 106). For *in vivo* applications, PEGylation (mostly with PEG-2000) on the liposome surface is commonly used (107). PEGylated liposomes acquire hydrophilicity on the surface, which might minimize the non-specific interaction with serum components by involving steric shielding and thereby increasing circulation time and overall stability of the NP (108). Some disadvantages of using PEGylated liposomes include the activation of immune responses and allergies (109). Furthermore, the PEG moiety hinders the interaction of liposomes with the target cell surface which could either decrease their cellular uptake, reduce their endosomal escape, and promote the degradation of the cargo in lysosomes (110). It is speculated that liposomes are accumulated in tumor tissues by passive targeting by virtue of an apparent tumor property known as “Enhanced Permeability and Retention” (EPR) effect *vs*. other tissues of the body, (110). The molecular and biological basis of the EPR are not well-understood (111).

Numerous studies have reported effective delivery of liposomes to glioblastoma cells. Ravi et al. used a liposomal formulation loaded with ferritin heavy chain 1 (FTH1)-targeted siRNAs to treat patient-derived xenograft glioblastoma initiating cells (GIC) (112). Kato et al. delivered (intratumorally) MGMT-targeted siRNAs loaded LipoTrust EX Oligo cationic liposomes in *s.c*. implanted Glioma-initiating cells (GIC)-derived tumors, and by osmotic pumps (*s.c*. implanted) in intracranially implanted GIC tumors (113). Their results showed that GIC tumors were sensitized to TMZ treatment (113). Our research team showed that a liposomal formulation of OMIs against miR-143 reduced tumor growth in a xenograft (*s.c*.) GBM mouse model (59). Despite their extensive use and their excellent biocompatibility, liposomes possess some disadvantages such as drug leakage and poor stability on storage leading to a short shelf-life (88). Although some siRNA-liposome formulations have advanced to clinical trials, and one to the clinic, none of them have been intended for brain tumors.

Polymeric NPs are solid-like colloidal particles synthesized with biodegradable polymers such as chitosan, collagen, or non-biodegradable polymers such as poly (lactic acid) (PLA) and poly (lactic-co-glycolic acid) (PLGA) having a size range of 50–300 nm,. It is speculated that compared with liposomes, polymeric NPs accumulate in cells of the target site due to their small size and penetration through capillaries. However, their synthesis is more complex than liposomes (88, 105) and additional optimization procedures are required. Danhier et al. used convention enhanced delivery (CED) (see section Viral-Based Delivery) to deliver EGFR- and galectin-targeted siRNAs as chitosan lipid nanocapsides to orthotopic (U-87 MG cells) GBM mouse models (114). They observed that the survival was increased significantly in mice treated with this formulation when compared with mice injected with each siRNA-chitosan formulation independently (114). Van Woensel et al. delivered a Galectin (Gal-1)-targeted siRNA-chitosan NPs intranasally to GBM mouse models (intracranially implanted GL261-WT or GL261-BFP tumor cells). Gal-1 which is overexpressed in GBM, was reduced by more than 50% as a result of this treatment (115). Intranasal delivery of the Gal-1-targeted siRNA-chitosan formulation in murine GBM models altered the tumor microenvironment, incremented CD4+ and CD8+ T cell numbers, sensitized cells to chemo and immunotherapy, and prolonged the animal survival (116). In other experiments, Ye et al. developed an angiopep-2-modified cationic PLGA nanoparticle to deliver Gefitinib (a tyrosine kinase inhibitor) and showed that this formulation effectively crossed the BBB and reduced the tumor growth in GBM mouse models (117).

Hybrids of lipids and polymeric particles have also been developed to deliver small molecules and siRNAs (118, 119). Dahlman et al. prepared a lipopolymer NP (LPNP) by conjugating epoxide-terminated lipids to low-molecular-weight polyamines (119). Yu et al. used this LPNP formulation to transfect siRNAs in brain tumor-initiating (BTIC) GBM cells (94). BTIC are present in brain tumors and possess stem cell properties including self-renewal and differentiation capacity into neural lineages (120). Moreover, BTIC are involved in tumor initiation, recurrence, and therapy resistance (94). BTIC are small in numbers relative to GBM cells, but their eradication is challenging as they survive chemotherapy and radiotherapy; they also reside in sites/conditions where therapies are less effective. Additionally, BTIC are heterogeneous in nature, stimulate epigenetic abnormalities, and rapidly migrate to normal cell sites to generate new GBM cell populations. However, targeting BTIC compared with other GBM cells could be a more effective approach for non-recurrent GBM treatment (87, 94). Yu et al. used LPNPs loaded with siRNAs against four transcription factors (SOX2, OLIG2, SALL2, and POU3F2); all responsible for proneural BTIC acquisition (94). Compared with non-targeted siRNA control NPs, intratumoral injection of siRNAs containing LPNPs increased the median survival of patient-derived BTIC xenograft in GBM mouse models (94). Likewise, Guerrero-Cázares et al. developed biodegradable poly (β-amino esters) (PBAEs) to deliver DNA in to BTIC (87). PBAEs are considered advanced cationic NPs which have been successfully used for gene delivery (121). The ester bonds of PBAE are readily cleaved by hydrolysis with effective release of the payload with minimum cytotoxicity (89). Green et al. used PBAE NPs to deliver miRNA mimics of miR-148a and miR-296-5p in orthotopic human GBM xenograft mouse models (89). Intratumoral administration of these NPs inhibited tumor growth and prolonged the survival of the animals (89, 122, 123). More recently, the same research group showed that the delivery of various siRNAs (against Robol, YAP1, NKCC1, EGFR, and survivin) within the same PBAE nanoparticle caused GBM cell death and reduced GBM cell migration (96). Intratumoral administration of this formulation reduced tumor burden in a xenograft (SC) GBM mouse model (96). More studies in relevant orthotopic GBM mouse models are needed to confirm that systemic administration of all of these NPs are able to cross the BBB and deposit their cargo in the tumor cells.

### Bacterial Toxins

Bacterial toxins (specifically AB-type toxins), such as anthrax toxin (AT) and diphtheria toxin (DT) or Pseudomonas exotoxin have been tested as promising tools for RNAi delivery (124). As these toxins have natural mechanisms to penetrate the cells, they can be further modified for drug delivery purposes (124–126). Besides, their distinctive structure allows their natural transport via receptor-mediated endocytosis and crossing the endosomal membranes via a transmembrane pore (124). When detoxified DT chimeras were used as protein-delivery vectors, high translocating efficiencies of cargo proteins of variable sizes (>100 kDa), structural motifs, and diverse stabilities were obtained (125). Dyer et al. successfully delivered Syntaxin5 (Synt5)-targeted siRNA in various primate cell lines (HeLa, THP-1 and Vero) using deactivated AT. Following transfection, a significant downregulation of Synt5 expression was observed compared with siRNA transfection with the nucleofection methods (126). Arnold et al. explored whether attenuated diphtheria toxin (aDT) could be used as a delivery vehicle for siRNAs against integrin-β1 (ITGB1)- and eukaryotic translation initiation factor 3 subunit b (eIF-3b)-targeted siRNAs in patient-derived GBM cells. First, they conjugated DT with cysteine (Cys); next, the Cys was modified with a maleimide cross-linked to dibenzocyclooctyne (DBCO) group and the siRNA molecules were attached to the DBCO. When the conjugated DT was added to GSCs, significant reductions in the mRNA levels of ITGB1 and eIF-3b were observed (127). More studies using bacterial toxins for RNAi delivery should be performed in GBM cells and GBM mouse models.

### Stem Cell-Derived Exosomes

Besides their use in prognosis, exosomes have gained potential use as drug delivery vehicles (128, 129). Compared with other NPs, exosomes exhibit less immunogenicity when derived from autologous cells and therefore they are less toxic compared with other artificial delivery vehicles (130). Due to the phospholipid bimolecular layer, exosomes can cross the plasma membranes (131). Their small size can facilitate their extravasation and diffusion in tumor tissues which can facilitate their transport across the BBB. Katakowski et al. used mesenchymal stromal cell (MSCs)-derived exosomes as carriers for miRNA delivery. They transfected MSCs with miR-146b-expressed plasmid and the isolated exosomes were injected intracranially in a xenograft rat model. The miR146b-exosomes reduced the tumor volume 5 days after the treatment (132). In another study, secreted exosomes were obtained from patient-derived GSCs engineered to express the miR-302-367 (133). Exosomes were delivered into naïve neighboring GSCs and resulted in repression of target genes, CXCR4/SDF1, SHH, cyclin D, cyclin A, and E2F1. The orthotopic xenograft of both naïve and ectopically expressing miR-302-367 GSCs altered the tumor development in mouse brain (133). The microRNA cluster miR-302-367 has previously been considered as a potential treatment for glioblastoma (134).

In a similar study, Lee et al. utilized MSCs to deliver miR-124 mimics in glioma xenograft mouse models. They transfected the MSCs with Cy3-labeled miR-124 mimics and administered them intracranially in the U-87-MG derived xenograft GBM mouse model (135). The results indicated that MSCs were able to deliver the synthetic exogenous miRNA mimics to glioma cells and glioma stem cells. However, the therapeutic potential of the formulation was not evaluated (135). Wang et al. studied the inhibitory effect of human bone marrow-derived mesenchymal stem cell (hBMSC)-derived exosomes in GBM tumor progression. They observed that hBMSC-derived exosomes overexpressing miR-34a suppressed MYCN expression and decreased cell proliferation, invasion, and migration; it reduced tumor growth in xenograft mouse models (136). Yu et al. have observed the inhibition of glioma progression by downregulation of AGAP2 (ArfGAP with GTPase domain, ankyrin repeat and PH domain 2) when exosomes were released from miR-199a-overexpressing MSCs *in vitro* and *in vivo* experiments (137). Despite the enormous potential of exosomes as RNAi carriers, additional *in vivo* experiments using orthotopic GBM mouse models should be performed.

### Viral-Based Delivery

Viral vectors including retrovirus, adeno-associated virus (AAV), and herpes simplex virus (HSV) have been studied for several years as a tool for gene therapy and as drug delivery vehicles (138–140). Lentiviral vectors have also emerged as efficient delivery vehicles for stable gene expression of shRNA in GBM cells (141). Yang et al. used lentivirus-based siRNA delivery to target small supernumerary marker chromosomes 1A (SMC1A). This gene plays an important role in genome stability including DNA replication, repair, and engaging proteins of cell-cycle networks (142). After infection of GBM cells with lentivirus containing SMC1A-targeted siRNAs, a 50 and 100% reduction in cell proliferation were obtained at 5 and 14 days, respectively (142). Matsuda et al. performed a remarkable study by developing a Hemagglutinating virus of Japan envelope (HVJ-E) which was obtained with inactivated Sendai virus. HVJ-E could be used as a delivery vector of siRNA, DNA, proteins, and other anti-cancer drugs (143). In this study, siRNA was incorporated in the HVJ-E vector to knockdown mitotic motor protein Eg5, which has important functions in the early stages of mitosis, centrosome separation, and formation of the bipolar mitotic spindle (143). Therefore, knockdown of Eg5 is beneficial as it is able to arrest the cell in the mitotic stage and leading to apoptosis. One of the most significant outcomes of this study was the complete eradication of all the intradermal U-118MG tumors and approximately 80% of intracranial implanted U-118 MG tumors (143).

In another study, epidermal growth factor receptor (EGFR) gene silencing was performed in human gli36-Luc glioma cells. HSV-1-based amplicons expressing EGFR specific siRNAs against two different locations pHSVsiEGFR I and pHSVsiEGFR II, respectively, were constructed. The knockouts at both loci resulted in a significant growth reduction of glioma cells *in vitro* and *in vivo* studies (144). HSV-1 vector was also employed in post-transcriptional inhibition studies of Rad51 protein in gli36-luc glioma cells containing target-specific siRNA combined with radiation therapy. Rad51 performs an important function in homologous strand exchange, which is a key step in DNA repair through homologous recombination (HR). Results of this study confirmed that the silencing of Rad51 improves the radiation-induced death of tumor cells (145). In another study, a recombinant adeno-associated virus (rAAV) expressing short hairpin RNA (shRNA) was produced in the vertebrate Spodoptera frugiperda (Sf9) cell line. This vector reduces the expression of Hec1 protein (highly expressed in cancer 1) which is responsible for the beginning of the anaphase by regulating chromosomal segregation, microtubule interactions, and spindle checkpoint signaling. When the vector (rAAV-shHec1) was injected intratumorally it selectively killed mitotically active glioma tumor cell (U251) in the xenografts tumor model (146). Lee et al. developed three-way-junction (3WJ)-based RNA NPs (RNP) derived from a well-established bacteriophage phi29 packaging RNA (pRNA) system. These bacteriophage-derived NPs were resistant to clinically relevant doses of I-125 and Cs-131 radiation (147). Further studies with multi-valent folate (FA)-conjugated 3WJ RNP loaded with LNA against miR-21 reduced the expression of this miRNA and incremented the expression of its target genes (PCDD4 and PTEN) in GBM cells *in vitro* and *in vivo* (148).

**Figure 1:**
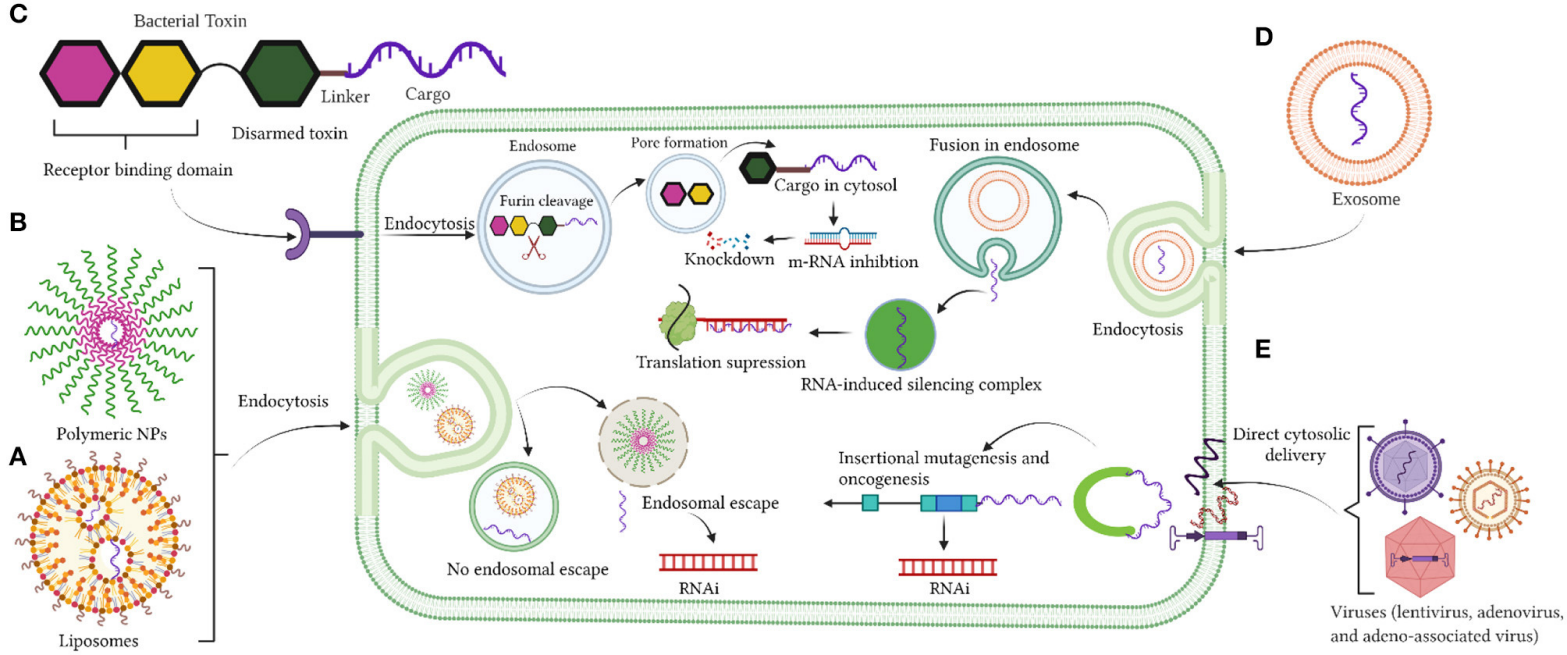
**(A–E)** Delivery strategies by liposomes nanoparticles, polymeric nanoparticles, bacterial toxin, exosomes, and viruses. Created with BioRender (https://Biorender.com/).

Despite the significant advancement with the use of viral vectors as cytotoxic agents and/or for gene therapy, clinical trials have failed (149–151). None of the proposed viral vectors against GBM have been approved clinically (152, 153). Nevertheless, a new category of AAV vector associated with exosomes, which are termed as vexosomes, has been reported (150, 154) and their applications were studied recently (149, 150, 154). These vectors were efficiently transduced and were more resistant in neutralizing anti-AAV antibodies compared to conventionally purified AAV (149). Although these vectors have not been investigated as delivery carriers for GBM treatment, they were very efficient in delivering DNA to the central nervous system (CNS) in mouse studies (150). Vexosomes were also used to deliver inducible caspase 9 (iCasp9) in Huh7 hepatic cancer cells and in hepatocellular carcinoma (HCC) xenograft mouse models (154). More exploratory studies are needed with the use of Vexosomes as drug delivery vehicles. Figures 1A–E summarizes the current delivery strategies under investigation for drug delivery into the brain.

## The Blood-Brain Barrier is the Critical Factor for the Successful Design of Useful Drugs for GBM Treatment

**Figure 2:**
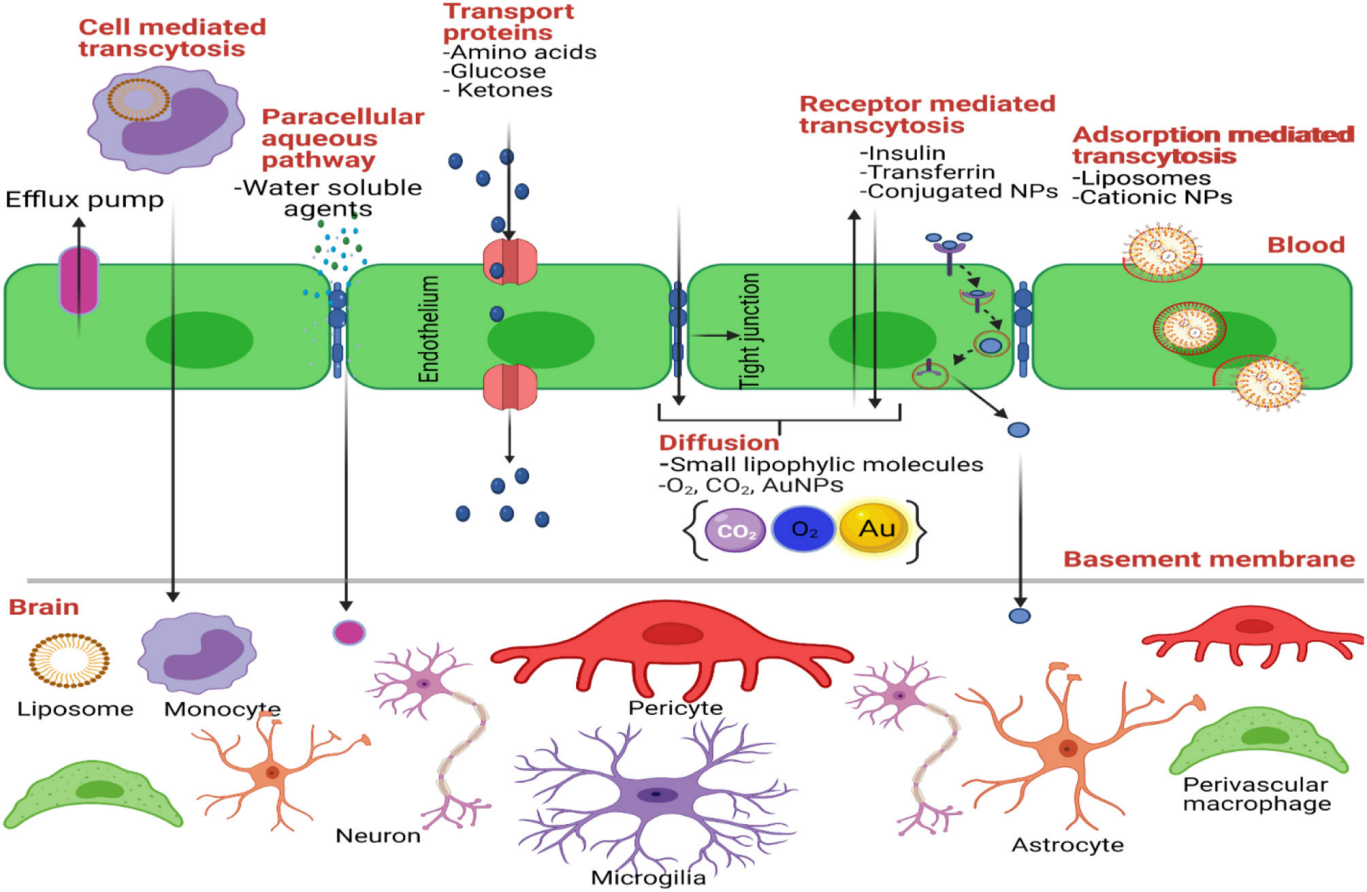
Transport mechanisms through the BBB. Created with BioRender (https://Biorender.com/).

The Blood-Brain Barrier (BBB) is the physical/anatomical barrier that protects and regulates the homeostasis of the Central Nervous System (CNS) (Figure 2). It is located between the blood microcirculation system and the brain parenchyma. The BBB is composed mainly of a non-fenestrated layer of brain microvascular endothelial cells, which are tightly bound together by Tight Junctions (TJ), surrounded by a specialized basal lamina that is shared with pericytes and astrocytic endfeet, and interconnected by neural endings and microglia (155, 156). TJ's, known also as Zona Occludens, contain major integral membrane proteins such as claudins and occudins. These proteins interact with peripheral membrane proteins known as Zonula Occludins (ZO) in order to maintain the integrity of the BBB. This integrity allows the BBB to be highly selective and regulate the entry of nutrients, ions, and other molecules. It also protects the CNS against neurotoxic substances. Likewise, pericytes are key regulators of vascular function throughout the body as they communicate with astrocytes and support BBB maintenance in the postnatal brain. Pericytes also regulate the expression of transporters such as the lysophosphatidylcholine (LPC) transport catalyzed by the Na^+^-coupled LPC symporter 1 (NLS1) which is responsible for the transport of docosahexaenoic acid (DHA, an omega-3 fatty acid) across the BBB (157). Pericytes enfold the CNS endothelium which forms a basal lamina that attracts astrocytic endfeet during development (158, 159). Astrocytes, the most abundant cell type in the brain, are important metabolic sensors and make a significant contribution to BBB development and function (160). Astrocytes regulate signaling pathways that maintain junctional complexes and produce an additional barrier called the glia limitans (161, 162). Together, astrocytes and pericytes play an important role in the integrity of the BBB (163).

Generally, the BBB precludes brain entry of 100% of large molecules and 98% of small molecules (164, 165). Although the BBB is compromised in GBM patients, this barrier is considered the rate-limiting factor for the development of all new therapeutics for the treatment of GBM; and other neurological disorders (163). Thus, research efforts are focused on finding ways to breach, bypass, and target the BBB to successfully develop optimal treatments against CNS-related conditions. Breaching the BBB consists of disrupting the neurovascular junction by chemical or mechanical insults, allowing treatments to enter the brain parenchyma. One example of this is the focused ultrasound-induced BBB opening (FUS-BBB) (166). Here, microbubbles are administered into the circulation and a non-invasive FUS is performed in the brain region of interest. When submitted to the low-energy toned ultrasound, the microbubbles burst and transiently permeate the BBB, causing a physical cavitation effect. This technique is being evaluated for the treatment of Alzheimer's disease (AD), amyotrophic lateral sclerosis (ALS), Parkinson's disease, and GBM (166). Due to successful preclinical studies in GBM, FUS-BBB is being evaluated under Phase I clinical trials for chemotherapy with doxorubicin and carboplatin (167).

Bypassing the BBB consists on finding new ways to deliver CNS treatments to the brain without dealing with the BBB. One way of achieving this is trading the oral, the intravenous (*i.v*.) or the intraperitoneal (IP) administration with non-conventional routes such as convection-enhanced delivery (CED), delivery to the cerebrospinal fluid (CSF) via intrathecal (IT) or intraventricular (IVN) routes, and intranasal delivery (168). Direct intratumoral drug administration and subcutaneous (*s.c*.) implantation of osmotic pumps have also been used for drug delivery using GBM mouse models (54, 55). In CED a pressure gradient is generated in a tip of an infusion catheter implanted in the interstitial spaces of the CNS. CED was used to deliver chitosan-siRNA NPs in GBM mouse models (114). Kim et al. implanted U-87 MG cells in the brain of mice and compared the tumor distribution of OMI (targeting Let-7) administered intratumorally (IT), intrathecally (ITc) and intraventricularly (Ivn). IT administration led to a high rate of OMI accumulation in the brain tumor cells (169). However, Ivn administration showed a greater distribution of the OMI in the brain tissues (169). Seo et al. used an orthotopic GBM mouse model (intracranial cell implantation) to deliver by CED NPs formulations loaded with miR-21 inhibitors (170). This treatment prolonged the survival of mice when combined with chemotherapy. Although CED has been used in preclinical and clinical studies, this procedure constitutes a very invasive method of drug delivery. Preclinical and clinical trials using CED to treat GBM and other brain-related conditions have not produced the expected results (171–173).

Another approach to bypass the BBB is intranasal (IN) drug delivery. IN delivery has been used to deliver chitosan-siRNA NPs in murine GBM mouse models (174). Sekerdag et al. administered (IN and *i.v*.) farnesylthiosalicylic acid (FTA) loaded (lipid-cationic) lipid-PEG-PLGA hybrid NPs in the brain of rat glioma (RG2) cell-bearing rats (175) and observed a 55.7% reduction in tumor area. Although both, *i.v*. and IN administration routes had a significant anti-cancer effect *in vivo*, IN delivery might be a better choice of treatment due to its low plasma levels and its non-invasive methods and lower systemic side-effects (175). More studies in GBM mouse models are needed before proposing IN drug administration as a real method to treat brain tumors. Additional procedures to optimize IN RNAi-NP formulations are also required.

A less invasive approach to cross the BBB is the systemic drug administration (Figure 2). For systemic administration, drugs should be modified to cross the BBB and reach the tumor cells in the brain. These modifications could allow drugs to cross the BBB using natural transport routes. Transport through BBB includes passive diffusion, paracellular trafficking, facilitated transport, adsorptive endocytosis, and/or receptor-mediated transcytosis (176). First, a drug that crosses the BBB through passive diffusion (lipid-mediated transport) needs to be small (<400 Da) and it must have high lipid solubility (<7 hydrogen bonds with solvent water) (177). For example, TMZ crosses the BBB since it has a molecular weight of 194 Da and it is highly lipophilic. One study revealed that of the >6,000 drugs in the Comprehensive Medical Chemistry database, only 6% are active in the brain (178). Second, small molecules could reach the brain paracellularly, between brain capillary endothelial cells. Under normal physiological conditions, there is no paracellular pathway from blood to the brain (179). This is because adjacent endothelial cells are cemented together through tight junctions, giving the BBB its high resistance property (180). However, in GBM and other neurological disorders, paracellular trafficking of small hydrophilic compounds can occur because the BBB is compromised (163). Also, new imperfect blood vessels are formed in GBM tumors, making the BBB leaky. Hence small NPs can be retained in the tumor tissue by the EPR (181). In addition to structural or architectural vascular abnormalities, impairment of lymphatic drainage and permeability enhancing factors contribute to the EPR effect (111, 182–184). In fact, a number of vascular mediators are utilized to augment the EPR effect. One of these mediators is Nitric Oxide (NO), an endogenous mediator that causes vessels to dilate and lower blood pressure, thus promoting EPR (181). A recent study by Yasuda et al. demonstrated that the use of NO-releasing agents has enhanced effects on cancer therapy (185).

Third, therapies can be modified to enable facilitated transport, also known as carrier-mediated transport (CMT), across the BBB. CMT comprises stereospecific pore-based transporters localized in the blood side and the brain side of the brain capillary endothelial cells (164, 165). These are dependent on chemical/electrical gradients (mostly dependent on sodium) and require certain structural characteristics for their affinity (186). Some examples of carrier-mediated transporters are glucose, lactate, amino acid, and adenosine transporters (GLUT1, MCT1, LAT1, and CNT2, respectively). Many drugs against brain-related disorders are designed to cross the BBB by using CMT (187). For example, L-DOPA for Parkinson's disease and gabapentin an anti-epileptic drug crosses the BBB through the L-type amino acid transporter 1 (LAT1) (188). Melphalan, an anticancer drug used mainly against multiple myeloma and other cancers, including recurrent brain tumors, crosses the BBB via LAT1 (189). Another anticancer agent that expresses an affinity for the BBB LAT1 transporter is Buthionine Sulfoximine (BSO). BSO is a glutathione synthesis inhibitor that has been used to enhance alkylating drug cytotoxicity and limit development of drug resistance (187). Additional drugs which cross the BBB via CMT should be designed and tested using brain tumor mouse models.

Fourth, effective therapeutic agents could penetrate the brain through adsorptive mediated endocytosis. Adsorptive mediated endocytosis is a vesicle mediated transport system that occurs upon non-specific interactions between positively charged macromolecules and the cell membranes of all cells, including brain capillary endothelial cells (190). Cationic NPs and other drugs conjugated to cell penetrating peptides (CPPs) can enable this type of transport in GBM. CPPs are short peptides derived from a protein-transduction domain that have the ability to enter into most type of cells and promote the delivery of conjugated biomolecules (191). Since most peptide- and nucleic acid-based drugs are poorly taken up by cells, the use of conjugated therapeutic agents to CPPs has become a subject of interest in improving drug delivery. Although the mechanisms of internalization of CPPs are not well-understood, recent studies have demonstrated that CPPs does not involve endocytosis nor specific protein transporters, but rather a possible direct transport through the lipid bilayer of membranes (192–194). Studies by Lakkadwala et al. evaluated the influence of inserting a CPP (TAT or QLPVM) in brain targeted with doxorubicin containing liposomes (195). They showed that CPPs significantly improved the systemic delivery of these liposomes across the BBB into glioblastoma tumor cells. Results of this study also demonstrated a higher accumulation of doxorubicin in the mouse brains as compared to free drug without toxicity (195). Although CPP exhibit great delivery potential to send drugs across the BBB, most CPPs are not cell specific, which limits their application in drug delivery.

Fifth, targeting brain capillary endothelial cells could improve drug delivery into brain tumors. Receptor mediated transcytosis (RMT) is one of the most promising mechanisms for systemic delivery for GBM treatment. Here, either small or large molecules can bind to specific receptors on the luminal (blood side) surface of capillary endothelial cells and cross the BBB by transcytosis mechanisms (196). Once macromolecules interact with their target receptor, they are internalized by endocytosis, transported across the cell's cytoplasm, and externalized by exocytosis from the abluminal (brain side) surface of capillary endothelial cells (197). In particular, peptides of apolipoprotein E (ApoE) which bind to low density lipoprotein (LDL) Receptor (198), angiopep-2 which binds to the receptor-related protein 1 (LRP-1) (199), hyaluronan (HA) which binds to CD44 receptor, and transferrin which binds to transferrin receptor have been used (200). For example, Gutkin et al. prepared hyaluronan (HA)-grafted lipid NPs (LNPs) loaded with Polo Like Kinase 1 (PLK1)-targeted siRNA. HA binds to CD44 receptor variant-containing cells (201). Intracranial administration of this formulation in tumor bearing mice (U-87 MG cells implanted intracranially) reduced PLK1 protein levels and prolonged the survival of the mice. Also, the LNPs were accumulated in the tumor tissue when they were systemic administered in C57Bl/6 mice (intracranially implanted with GSCs). Published data from Böckenhoff and collaborators demonstrated that ApoE, in comparison to other BBB permeable therapeutic polypeptides including Angiopep (Ang-2), Apolipoprotein B (ApoB), and Transactivator of Transcription (TAT) exhibited the highest brain accumulation of the lysosomal enzyme Arylsulfatase A (ASA) (202). Studies by Fu et al. demonstrated that incorporating a glucose-RGD derivative into paclitaxel containing liposomes for dual targeting [via GLUT1 (glucose) and integrin αvβ3 (RGD)] increased liposome accumulation in Kunming mice bearing C6 glioma tumors compared to paclitaxel alone (203).

Saw et al. developed aptamer-like peptide (aptide)-decorated liposomal nanoplatform for siRNA delivery into GBM cells. The NPs were decorated with PEG and with a surface-encoded aptide to precisely target the extra-domain B (EDB) of fibronectin, a glycoprotein overexpressed on glioma cells. They used siRNA to target cyclophilin A (CypA), a gene upregulated in brain cancer cells that plays a critical role in malignant transformation and maintenance of glioma cell stemness (204). This aptamer NP formulation decreased cell growth *in vitro*, and when administed IV. reduced tumor growth in a s.c. GBM mouse model. Recently, Zou et al. used an angiopep-2-decorated nanocapsule with PLK1-targeted siRNA inside (205). Systemic administration of this NPs formulation in an orthotopic GBM mouse model (intracranial implantation of U-87 MG cells) increased their circulation in plasma and their accumulation in the GBM cells (205).

Another modification to improve systemic treatments against CNS disorders is the conjugation of drugs with peptide sequences from neurotropic viruses—capable of invading and infecting neural tissue. Some viruses, like the West Nile virus, are able to get through the BBB by infecting immune cells that enter the CNS (206), while others like the ZIKA virus have shown to directly infect the BBB endothelial and glial cells (207). However, most viruses enter the CNS through the peripheral nervous system (PNS), using the nerve tracts to transit from the periphery to the CNS, evading the BBB (208). The rabies virus is an example of a neurotropic virus that uses axonal transport (209). This virus contains a rabies virus glycoprotein (RVG) which is the part of the virus responsible for neural interaction and which has been used for drug delivery to the brain (210). Evidence indicates that RVG interacts specifically with the nicotinic acetylcholine receptor (nACnR) in neural cells (211–214). Early studies by Kumur et al. showed that RVG peptide significantly increased oligonucleotide delivery (siRNA) to brain (*p* = 0.001) in comparison to other organs (liver and spleen), making it an excellent candidate to improve RNA delivery to brain tumors (212). In a recent study, our research team compared the accumulation levels of RVG- and ApoE-decorated liposomal nanoparticles (with AuNPs-OMI inside liposomes) in orthotopic GBM mouse models (106). We showed that ApoE decorated NPs accumulate to a higher degree in GBM cells compared with RVG-decorated NPs (106). In a separate study, Kong et al. prepared arginine-glycine-aspartic (RGD) functionalized dendrimer-entrapped AuNPs (Au DENPs) and polyethylene glycol (PEG) spacers attached to siRNA molecules against vascular endothelial growth factor (VEGF) and B-cell lymphoma/leukemia-2 (Bcl-2). Effective silencing of both, VEGF and Bcl-2 was observed in both *in vitro* and *in vivo* studies (215, 216). Similar results were obtained when dendrimers were replaced by polyethyleneimine (PEI) (217).

Molecular Trojan horse (MTH) is an engineered endogenous peptide monoclonal antibody (MAb) that can undergo receptor mediated transport across BBB (218). Several species-specific Mab MTHs have been developed for brain drug delivery (219). In humans, a genetically engineered form of human insulin receptor (HIR) Mab have been produced to enable drug delivery into the brain. RNAi-based molecules can be attached to MTH by avidin-biotin conjugation (219). This attachment has no effect on the hybridization of the RNAi molecule with its RNA target (219). A combination of MTH and liposomes, called Trojan Hourse Liposomes (THL), have been used for siRNA delivery. Zhang et al. implanted U-87 MG cells in the brain of nude mice and 5 days later injected (*i.v*.) a EGFR-targeted short hairpin RNA loaded PEGylated-liposomes. Liposomes were derivatized with two monoclonal antibodies, a murine MAb to the human insulin receptor and a rat MAb to the mouse transferrin receptor. Weekly administration of this THL increased mouse survival by 88% while EGFR expression was significantly reduced. Other THL formulations for drug delivery into the brain are being developed (220).

## Conclusions and Future Remarks

Currently, an arsenal of technologies for GBM diagnosis are available in the clinical setting. Also, the classification of CNS tumors according to pathological and molecular parameters is an advantage as more precise GBM diagnostic and prognostic tools are now possible. Although many therapies for GBM treatment are in the clinic and in clinical trials, GBM is still an incurable and deadly disease. Deregulation of several miRNAs and lncRNAs have been reported in GBM cell lines and GBM tumor samples and they represent promising targets against GBM. Given the nature of miRNA binding to multiple mRNAs, the precise molecular and biological consequences of targeting a miRNA should be carefully studied. These studies should be conducted not only in the tumor cells but also in tumor microenvironment. Regarding the lncRNAs, more studies are needed to fully understand the role of these regulatory and signaling molecules in health and disease. Targeting some miRNAs and lncRNAs with RNAi molecules in GBM cell lines and GBM mouse models have resulted in beneficial effects. However, the delivery of RNAi molecules to the brain is a challenge as BBB precluded the passage of most substances into the brain.

Biodegradable NPs are ideal carriers to delivery RNAi molecules to GBM tumors. IN and *i.v*. delivery are the least invasive routes to deliver RNAi-NPs (and other drugs) to the brain. Although thousands of NPs have been suggested for the delivery of RNAi molecules for GBM treatment, few have been tested in animal models. Some reports have shown that IV delivery of RNAi-NPs cross the BBB (by RMT) and efficiently accumulate in the tumor cells of orthotopic GBM mouse models. More studies implanting patient derived GBM tumors and/or GBM cell lines in the mouse brain (intracranial, orthotropic) are required. Also, to model a genuine treatment, the therapy should be administrated IV or IN. Although, the use of SC GBM mouse models to test the therapeutic benefits of a novel RNAi-based therapies is a first approach, this does not represent a real GBM model, mainly because the tumor microenvironment and the presence of BBB are not considered. Designing NPs able to discriminate tumor *vs*. normal cell is another critical challenge to overcomes. Therefore, multidisciplinary research teams should work together in order to design rational NPs able to cross the BBB. In this way the RNAi-based therapies for GBM treatment will become a clinical reality.

## Author Contributions

PEV-M, RKS, and CC wrote sections of the manuscript. RKS elaborated the figure of the manuscript. PEV-M made corrections to the final version of the manuscript. All authors contributed to manuscript revision, read, and approved the submitted version.

## Conflict of Interest

The authors declare that the research was conducted in the absence of any commercial or financial relationships that could be construed as a potential conflict of interest.

## Publisher's Note

All claims expressed in this article are solely those of the authors and do not necessarily represent those of their affiliated organizations, or those of the publisher, the editors and the reviewers. Any product that may be evaluated in this article, or claim that may be made by its manufacturer, is not guaranteed or endorsed by the publisher.
